# Fine-Tuned Large Language Models for Generating Multiple-Choice Questions in Anesthesiology: Psychometric Comparison With Faculty-Written Items

**DOI:** 10.2196/84904

**Published:** 2026-02-18

**Authors:** Carlos Ramon Hölzing, Charlotte Meynhardt, Patrick Meybohm, Sarah König, Peter Kranke

**Affiliations:** 1 Department of Anaesthesiology, Intensive Care, Emergency and Pain Medicine, University Hospital Würzburg, Oberdürrbacher Str. 6, Würzburg, 97080, Germany; 2 Institute of Medical Teaching and Medical Education Research, University Hospital Würzburg, Würzburg, Germany

**Keywords:** medical education, multiple-choice questions, large language models, fine-tuning, psychometrics, assessment, anesthesiology, artificial intelligence

## Abstract

**Background:**

Multiple-choice examinations (MCQs) are widely used in medical education to ensure standardized and objective assessment. Developing high-quality items requires both subject expertise and methodological rigor. Large language models (LLMs) offer new opportunities for automated item generation. However, most evaluations rely on general-purpose prompting, and psychometric comparisons with faculty-written items remain scarce.

**Objective:**

This study aimed to evaluate whether a fine-tuned LLM can generate MCQs (Type A) in anesthesiology with psychometric properties comparable to those written by expert faculty.

**Methods:**

The study was embedded in the regular written anesthesiology examination of the eighth-semester medical curriculum with 157 students. The examination comprised 30 single best-answer MCQs, of which 15 were generated by senior faculty and 15 by a fine-tuned GPT-based model. A custom GPT-based (GPT-4) model was adapted with anesthesiology lecture slides, the National Competence-Based Learning Objectives Catalogue (NKLM 2.0), past examination questions, and faculty publications using supervised instruction-tuning with standardized prompt–response pairs. Item analysis followed established psychometric standards.

**Results:**

In total, 29 items (14 expert, 15 LLM-generated) were analyzed. Expert-generated questions had a mean difficulty of 0.81 (SD 0.19), point-biserial correlation of 0.19 (SD 0.07), and discrimination index of 0.09 (SD 0.08). LLM-generated items had a mean difficulty of 0.79 (SD 0.18), point-biserial correlation of 0.17 (SD 0.04), and discrimination index of 0.08 (SD 0.11). Mann-Whitney *U* tests revealed no significant differences between expert- and LLM-generated items for difficulty (*P*=.38), point-biserial correlation coefficient (*P*=.96), or discrimination index (*P*=.59). Categorical analyses confirmed no significant group differences. Both sets, however, showed only modest psychometric quality.

**Conclusions:**

Supervised fine-tuned LLMs are capable of generating MCQs with psychometric properties comparable to those written by experienced faculty. Given the limitations and cohort-dependency of psychometric indices, automated item generation should be considered a complement rather than a replacement for manual item writing. Further research with larger item sets and multi-institutional validation is needed to confirm generalizability and optimize integration of LLM-based tools into assessment development.

## Introduction

Multiple-choice questions (MCQs) are fundamental to the objective assessment of medical students. They allow standardized testing across large cohorts and play a central role in evaluating foundational and applied knowledge [1]. However, the development of high-quality MCQs demands not only deep domain knowledge but also significant methodological and didactic expertise [2,3]. Effective items must balance appropriate difficulty, plausible distractors, minimal cueing, and strong discriminatory power to differentiate between varying levels of student performance [4].

Recent advances in artificial intelligence (AI), particularly large language models (LLMs), offer novel tools for automated question generation. For instance, efforts comparing ChatGPT-3.5–generated MCQs with expert-written items in neurophysiology revealed similar difficulty levels but lower discriminatory power in LLM-generated questions [5]. A systematic review of LLM use in medical MCQ generation found that while LLMs can produce examination-relevant items, many require additional modification due to quality issues [6]. Other studies highlight linguistic and structural shortcomings in automatically generated MCQs, particularly regarding distractor plausibility and alignment with instructional content [7,8].

Recent domain-specific efforts such as Hypnos [9], CDGen [10], and the Chinese Anesthesiology Benchmark [11] have demonstrated that LLMs can be effectively fine-tuned or benchmarked within anesthesiology. However, these studies primarily focus on domain adaptation and benchmark performance rather than psychometric validation of automatically generated examination items. To address this gap, a GPT-based model was adapted using anesthesia-specific teaching materials, the National Competence-Based Learning Objectives Catalogue in Medicine (NKLM 2.0), past examination items, and faculty publications [12]. Item development for both expert- and AI-generated questions was systematically mapped to the NKLM 2.0, Bloom’s taxonomy, and the local examination blueprint to ensure comprehensive curricular coverage and to allow a fair psychometric comparison.

This study aimed to evaluate whether a fine-tuned LLM can generate MCQs (Type A) in anesthesiology with psychometric properties comparable to those written by expert faculty.

## Methods

### Overview

This study analyzed the performance of MCQs used in the regular written examinations of anesthesiology in the eighth semester of medical training with 157 students. The examination consisted of 30 items. Half of the items (n=15) were written by senior faculty members, and half (n=15) were generated by a fine-tuned LLM. Nine faculty members from the Department of Anesthesiology, each with at least 10 years of experience, participated in item creation. All had prior training in assessment design through institutional workshops on multiple-choice item writing. In addition, all items were independently reviewed by an educational specialist with a Master of Medical Education degree to ensure adherence to established item-writing principles. Faculty were aware of the study but blinded to the psychometric comparison during data collection.

Data analysis was performed fully anonymously. The participating students were regular medical students in their eighth semester. They were not informed about the origin of the examination questions and therefore did not know whether an item was generated by faculty or the LLM.

A customized GPT-based model was developed specifically for this study. The model was built as a domain-adapted instance of GPT-3.5-Turbo, configured to generate single-best-answer MCQs. Adaptation followed a supervised instruction-tuning approach: several hundred standardized prompt-response pairs were created using anesthesiology lecture slides, NKLM 2.0, past examination questions, and faculty publications. Faculty publications were included to capture authentic domain phrasing and ensure that the model reflected institution-specific conceptualizations of anesthetic procedures. Previous research shows that faculty development and the use of high-quality source material improve item validity and discrimination [13].

These materials were curated to align with Bloom’s taxonomy and national curricular requirements [14]. The fine-tuning pipeline can be found in Multimedia Appendix 1.

Item analysis followed established psychometric standards. Difficulty was defined as the mean proportion of correct responses (0‐1). Values between 0.30 and 0.70 are generally considered optimal, those greater than 0.70 indicate easy items, and those less than 0.30 indicate difficult items [15,16]. The point-biserial correlation was classified as follows: negative correlation (*r*<0), very low correlation (0≤*r*<0.10), low correlation (0.10≤*r*≤0.20), and acceptable correlation (*r*≥0.20) [15]. The discrimination index (D) was calculated as the difference in difficulty between the upper and lower 27% performance groups, with values of 0.40 and above considered excellent; 0.30‐0.39, good; 0.20‐0.29, acceptable; and those less than 0.20, poor [15,16]. Statistical analysis was performed using SPSS Statistics version 27 (IBM Corp). Graphs were created with Prism 9 (GraphPad Software). Nominal variables were summarized as counts and percentages. The Shapiro-Wilk test was used to test for normal distribution. Group comparisons of categorical data were performed with the chi-square test or Fisher exact test if expected frequencies were less than 5. Continuous data were reported as mean and SD values and compared using the Mann-Whitney *U* test. A significance level of *P*≤.05 was applied.

**Figure 1.**
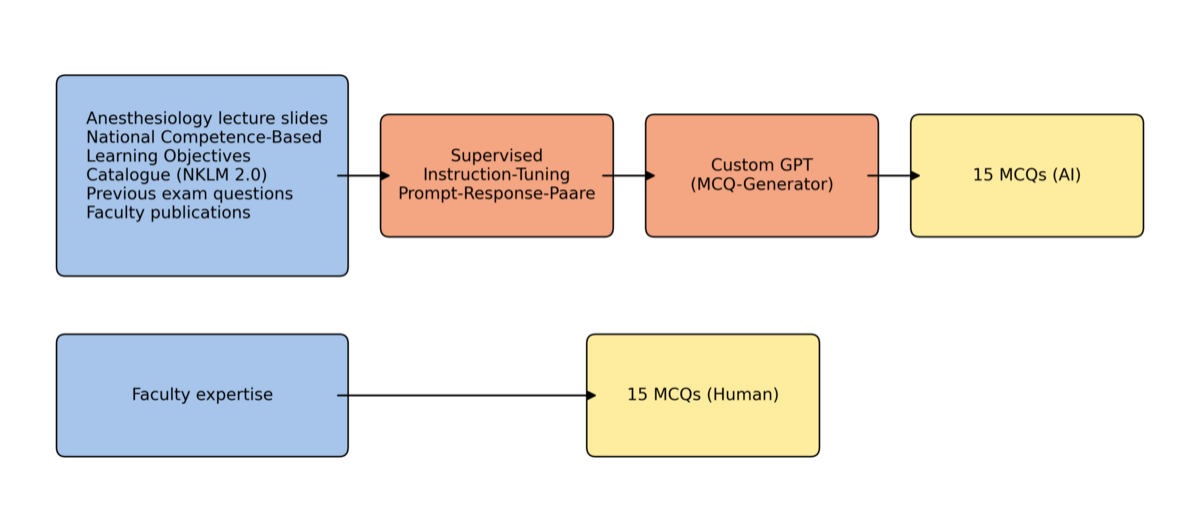
Item-generation workflow. Consolidated inputs (anesthesiology lecture slides, NKLM 2.0, prior examination items, faculty publications) inform supervised instruction-tuning of a custom GPT configured for single-best-answer MCQs to produce 15 AI-generated questions. In parallel, faculty authored 15 questions. AI: artificial intelligence; MCQ: multiple-choice question.

Figure 1 summarizes the item-generation workflow, including consolidated inputs, supervised instruction-tuning, the custom GPT MCQ generator, and parallel faculty-written items converging into the examination.

### Ethical Considerations

The study was submitted to the Ethics Committee of the University of Würzburg, which confirmed (reference number 2024-‐258-ka on November 11, 2024) that no formal review was required and that no ethical objections were raised.

## Results

A total of 30 MCQs were analyzed. One expert-generated item was excluded from analysis due to a strongly negative discrimination index (–0.22) and negative point-biserial correlation (–0.20). Its difficulty (*P*=.86) indicated a ceiling effect, suggesting that most students answered it correctly despite unclear key wording.

**Figure 2.**
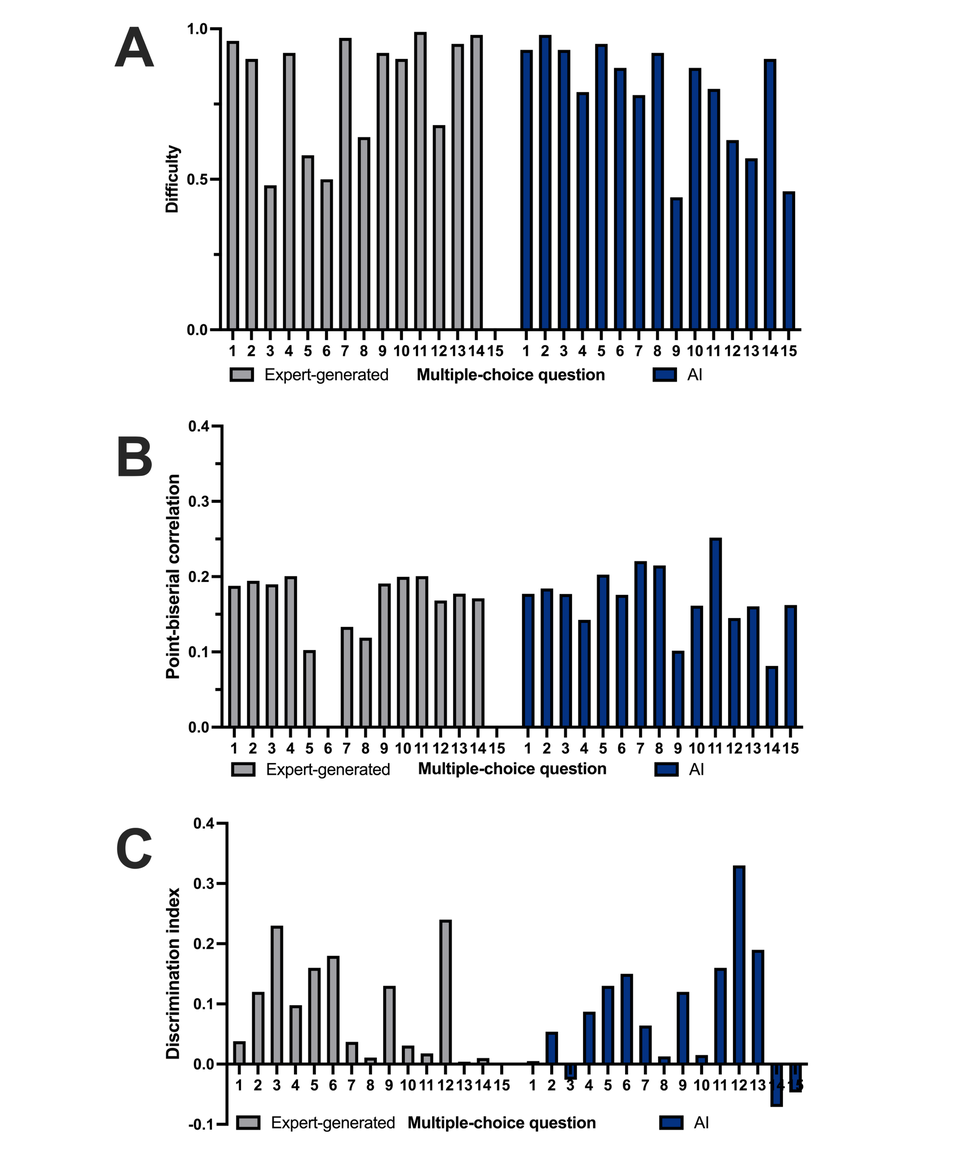
Psychometric characteristics of AI- and expert-generated multiple-choice items*.* (A) Item difficulty, (B) point-biserial correlation, and (C) discrimination index is displayed for each question*.* AI: artificial intelligence.

The final dataset therefore included 14 expert-generated and 15 AI-generated items. Table 1 displays the descriptive metrics for expert- and AI-generated items. Expert-generated items showed a mean difficulty of 0.81 (SD 0.19), a mean point-biserial correlation of 0.16 (SD 0.07), and a mean discrimination index of 0.09 (SD 0.08). AI-generated items had a mean difficulty of 0.79 (SD 0.18), a mean point-biserial correlation of 0.17 (SD 0.04), and a mean discrimination index of 0.08 (SD 0.11). Mann-Whitney *U* tests indicated no significant differences between expert- and AI-generated items with respect to difficulty (*P*=.38), point-biserial correlation (*P*=.96), or discrimination index (*P*=.59; Figure 2).

**Table 1.** Overview of question metrics by expert and artificial intelligence (AI).

Question created by	Questions, n	Metrics		
		Minimum	Maximum	Difficulty, mean (SD)
Expert				
Difficulty	14	0.48	0.99	0.81 (0.19)
Point-biserial correlation	14	−0.02	0.20	0.16 (0.07)
Discrimination index	14	0.01	0.24	0.09 (0.08)
AI				
Difficulty	15	0.44	0.98	0.79 (0.18)
Point-biserial correlation	15	0.08	0.25	0.17 (0.04)
Discrimination index	15	−0.07	0.33	0.08 (0.11)

Reference ranges are as follows:

- Difficulty (*P*)=0.30-0.70 is considered desirable;
- Discrimination (*r*_pb_)≥0.20 is considered acceptable;
- *r*_pb_<0 indicates flawed items [17].

The categorical distributions of item properties are summarized in Table 2.

**Table 2.** Psychometric characteristics of expert- and LLM^a^-generated items displayed side by side for difficulty, point-biserial correlation, and discrimination index.

	Questions, n (%)	
	Expert-generated (n=14)	LLM-generated (n=15)
Difficulty categories		
Extremely difficult (*P*≤0.25)	0 (0)	0 (0)
Tends to heavy (0.25<*P*≤0.4)	0 (0)	0 (0)
Optimal difficulty (0.4<*P*≤0.8)	5 (35.7)	6 (40.0)
Very simple (0.8<*P*≤0.9)	0 (0)	3 (20.0)
Extremely simple (*P*>0.9)	9 (64.3)	6 (40.0)
Point-biserial correlation categories		
Negative correlation (*r*<0)	1 (7.1)	0 (0)
Very low correlation (0≤*r*<0.10)	0 (0)	1 (6.7)
Low correlation (0.1≤r≤0.20)	8 (57.1)	10 (66.7)
Acceptable correlation (*r*≥0.20)	5 (35.7)	4 (26.7)
Discrimination index categories		
Urgent need for revision (D’<0)	0 (0)	3 (20.0)
Need for revision (0≤D’<0.2)	12 (85.7)	11 (73.3)
Check required (0.2≤D’<0.3)	2 (14.3)	0 (0)
Potential for improvement (0.3≤D’<0.4)	0 (0)	1 (6.7)
Item effectively distinguishes (D’≥0.4)	0 (0)	0 (0)

a LLM: large language model.

## Discussion

### Principal Findings

In this study, we compared psychometric properties (difficulty, point-biserial correlation, and discrimination) of MCQs generated by a supervised fine-tuned LLM with those written by expert faculty in an undergraduate anesthesiology examination. Although no statistically significant differences were observed, the overall quality of both item sets remained moderate. The point-biserial correlations and discrimination indices suggest that neither set reliably distinguishes higher- from lower-performing students, a finding consistent with previous research indicating that even expert-authored items often underperform in psychometric analyses [18]. This pattern aligns with broader evidence in medical education, where cohort studies have demonstrated that AI-generated MCQs often achieve discrimination indices similar to expert-generated items but tend to be easier overall and still require expert review to ensure distractor plausibility and alignment with higher-order learning objectives [5,19-23].

Supervised adaptation with domain-specific materials likely contributed to the close alignment of psychometric indices between AI and faculty-written items. Other work shows that when AI-mediated question generation is guided by domain content, structured prompts, or instruction tuning, the output more closely resembles faculty items in both difficulty and discrimination [7,8,19,24]. Notably, neither item set in our study consistently achieved high point-biserial correlation or discrimination, confirming that generating functionally effective distractors remains a challenge for both experts and LLMs [25-30]. Prior studies have similarly identified that AI items often underperform in assessing higher cognitive levels or using plausible distractors without ambiguity [8].

The absence of psychometric superiority in either group suggests that AI-assisted question generation can produce items of comparable statistical quality to traditional item writing. However, psychometric analysis alone is insufficient for examination quality assurance; human oversight remains essential to safeguard content validity, blueprint alignment, and cognitive level coverage. Studies in high-stakes examination settings show that expert review reduces factual inaccuracies and improves alignment with assessment blueprints [31]. Importantly, in our study, the fine-tuned LLM generated all 15 candidate items within a few minutes. While we evaluated only a subset psychometrically, our study demonstrates that domain-adapted LLMs support rapid item drafting at scale. Automatic item generation methods have long promised efficiency gains by expanding item pools from templates rather than crafting each item manually [32]. Recent AI studies show that LLM-based MCQ generation can approach human performance while drastically reducing human effort [33]. In practice, educators may use LLM throughput to generate large candidate sets and then filter, refine, and align items to the blueprint and cognitive levels, shifting effort from generation toward qualitative review and validation.

### Limitations and Future Work

Study limitations include a small item sample size, single-institution administration, and fine-tuning with primarily local teaching resources, which may reduce external validity. Cognitive level of items (eg, recall vs application) was not measured, although comparative studies indicate this is an important differentiator between AI- vs expert-generated MCQs [31]. Future work should involve larger item pools, multi-institutional validation, and systematic qualitative review of items, including stem clarity, distractor plausibility, and distractor efficiency, as well as cognitive demand. It would also be valuable to compare different fine-tuning or prompt-engineering strategies and to assess students’ perceptions of AI-generated items [34].

### Conclusion

This study demonstrates that a supervised fine-tuned LLM can generate MCQs with psychometric properties comparable to those created by experienced faculty. While neither approach consistently produced items with high point-biserial correlation or discrimination, the results indicate that automated question generation can complement traditional item writing in medical education.

## Supplementary material

10.2196/84904 Multimedia Appendix 1 Technical workflow and dataset construction for fine-tuned large language model–mediated item generation.

## Abbreviations

AI: artificial intelligence
LLM: large language model
MCQ: multiple-choice question
NKLM 2.0: National Competence-Based Learning Objectives Catalogue in Medicine
