# Supplementary material for: Fine-Tuned Large Language Models for Generating Multiple-Choice Questions in Anesthesiology: Psychometric Comparison With Faculty-Written Items
Source: JMIR Form Res. 2026 Feb 18;10:e84904. doi: 10.2196/84904 (PMC12916093; doi:10.2196/84904)
Supplement: Multimedia Appendix 1 [file formative-v10-e84904-s001.pdf]

Supplemental

Fine-Tuning Dataset Construction

A domain-specific training corpus was curated from four primary sources:

1. Anesthesiology lecture slides of the local 8th semester curriculum
2. NKLM 2.0 (National Competence-Based Learning Objectives Catalogue for Medicine), mapping each question to a unique NKLM learning objective ID
3. De-identified previous exam questions, reviewed to prevent direct item reuse and ensure novelty
4. Faculty-authored publications and clinical guidelines from the Department of Anaesthesiology, Intensive Care, Emergency and Pain Medicine, University Hospital Würzburg

Integration of guidelines and publications:
Relevant guideline recommendations and key statements from faculty publications were manually extracted and segmented into knowledge units. These units were annotated with topic, NKLM learning objective, and Bloom level and used as input in instruction - response pairs. This ensured that the model was not exposed to entire documents verbatim but rather learned how to generate exam-quality questions from structured, validated content snippets. This process also guaranteed traceability of each generated MCQ back to its curricular or evidence-based source.

Each knowledge unit was enriched with metadata:

- topic: domain-specific tag (e.g., “airway”, “hemodynamics”).
- bloom_level: cognitive level (recall, comprehension, application, analysis).
- learning_objective: NKLM ID (e.g., 07.3.1.1.3).

The final dataset was stored as JSONL containing standardized instruction-response pairs.

Example entry:

{
  "instruction": "Generate a single-best-answer MCQ testing application-level knowledge on airway management. Align with NKLM learning objective 07.3.1.1.3. Use a clinical vignette, ensure 1 correct answer and 4 plausible distractors. Return output as JSON with keys 'stem', 'options', and 'correct_option'.",
  "input": "Source material: Lecture slides on difficult airway, S1 guidelines on airway management (DGAI 2021). Learning goal: student can select the most appropriate airway device for a patient with Mallampati IV.",
  "output": {
    "stem": "A 65-year-old male presents for elective hernia repair. He has a Mallampati IV score and limited neck extension. Which airway device is most appropriate for securing the airway?",
    "options": [
      "Video laryngoscope",
      "Classic laryngeal mask airway",
      "Standard direct laryngoscopy",
      "Cricothyrotomy set",
      "Nasal cannula with high-flow oxygen"
    ],
    "correct_option": "Video laryngoscope"
  }
}

Fine-Tuning Pipeline

Fine-tuning was performed using the OpenAI managed fine-tuning API with supervised instruction-tuning.

Process details:

- Base model: gpt-3.5-turbo (instruction-tuned variant)
- Data split: 90% training / 10% validation
- User-exposed hyperparameters:
    - n_epochs grid: 3 and 5
    - batch_size: 8

- learning_rate_multiplier: 0.1 and 0.2

The best model was selected based on lowest validation loss. Optimizer, loss function, and weight update procedure are handled internally by OpenAI and are not user-configurable.

Item Generation Procedure

Item generation was performed via the OpenAI API using a low temperature (temperature = 0.2) to minimize variance and approximate reproducibility. A seed parameter was specified, though results remain only quasi-deterministic.

The example shown below represents a single prompt-response pair. In practice, a diverse set of prompts was used to cover different NKLM learning objectives and sources ensuring variability and reducing overfitting to a single question style.

```
import openai
openai.api_key = "YOUR_API_KEY"

prompt = """
Generate a single-best-answer MCQ for undergraduate medical students in
anesthesiology.
Requirements:
- Align strictly with NKLM 2.0 learning objective: 07.3.1.1.3 (Airway Management)
- Use a concise clinical vignette (2–3 sentences)
- Provide 1 correct answer and 4 plausible distractors (mutually exclusive)
- Avoid trivial cues or absolute terms (“always”, “never”)
- Return output strictly in JSON with keys:
  "stem": question stem,
  "options": list of 5 options,
  "correct_option": exact string match of the correct option
- Language: German, formal academic style
"""

response = openai.ChatCompletion.create(
```

```
    model="ft:gpt-3.5-turbo:anesth-mcq-2024",
    messages=[
        {"role": "system", "content": "You are a medical educator generating exam-
quality MCQs."},
        {"role": "user", "content": prompt}
    ],
    temperature=0.2,
    max_tokens=512,
    seed=42
)

print(response["choices"][0]["message"]["content"])
```

All generated MCQs were stored as structured JSON and underwent dual faculty review. Items with factual inaccuracies, ambiguous distractors, or insufficient alignment with NKLM objectives or guideline content were excluded or manually revised prior to inclusion in the exam.
